# Œdema of Scrotum and Penis, Etc.

**Published:** 1873-07

**Authors:** W. S. Armstrong

**Affiliations:** Atlanta, Ga., Late Professor of Anatomy in Atlanta Medical College


### ŒDEMA OF SCROTUM AND PENIS, OF AN ERYSIPELATOUS CHARACTER.

BY W. S. ARMSTRONG, M.D., ATLANTA, GA.,
*Late Professor of Anatomy in Atlanta Medical College.*

[Read Before the Georgia Medical Association, April, 1873.]

Several cases of the above disease having recently fallen under my care, I have thought proper to extract the history of one or more from my note-book, though I have had time to do it but imperfectly. I have determined to present them, too, for the reason that several winters ago I saw extensive sloughing of the scrotum—even to leaving the testes bare—in one of the hospitals of Europe, from this effusion into scrotum, and for the further reason that more recently I saw a partial slough in a case of my own. I feel fully convinced, therefore, that to conduct this affection to a safe result, the course adopted should be prompt and efficient, or a like result may be expected frequently to follow.

Œdema of the scrotum and penis usually results from an abrasion, a wound, a calculus, or some foreign body in the urethra, and in one case I saw, it arose from treating a stricture of the urethra by dilatation.

The first symptom noticed may be, as in other erysipelatous inflammations, a rigor, followed alternately by flushes and chills, nausea, vomiting, and furred tongue; or, the first sign may be the œdematous swelling, soon to be followed by constitutional disturbance.

In the outset the scrotum is of a scarlet hue, pits on pressure, and in a short time swells to a considerable size. It has a waxy and semi-transparent look. If not operated on, the scrotum becomes tense, and spots of extravasated blood may occur, due to the great swelling and obstruction of the vessels of the dartos, which threatens to slough. In some instances this does occur—even to leaving the testes exposed.

At the first glance, one might suppose it an acute hydrocele; but the history, together with the scarlet hue and the sense of touch, will enable him to determine the difference.

*Treatment.*—There is no palliative course of treatment to be used in this affection. There should be no waste of time in trying local applications and remedies. Under their use alone one would almost certainly be mortified by seeing the soft parts slough.

The scrotum must be relieved of the sero-plastic exudation, which distends it, and is so pressing upon the vessels of the dartos as to threaten the obliteration of their channels. Under these circumstances, then, the only remedy is to use the bistoury freely, in order to allow the serum to be discharged, and thereby relieve the tension. The sooner this is done the better will the result be.

The incisions should be one or more on each side of the raphe—they should be three or four inches in length—extending near the whole length of the scrotum from the perineum to the integument on the under surface of the penis. As the testicles lie at the upper and back part of the swelling, the incisions should be deep, which will allow a free and rapid discharge of the serous effusion and prevent a destruction of the soft parts by sloughing.

When incised, the walls or edges have a semi-transparent, gelatinous appearance, of a straw color. From these the sero-plastic effusion runs down in almost a continuous stream, and from these extensive cuts, strange to say, scarcely enough blood escapes, in some cases, to stain the serum that flows away.

I will only give a short and incomplete report of one or two cases of recent date, which, however, are a fair sample of others I have met with.

*Case 1.—Œdema of Scrotum and Penis—Treated by Incisions—Recovery.*

History.—G. B., aged two years. Saw him at 8 A.M., March 4, 1873. From his family learned that for four or five days he had seemed uneasy, and complained of pain while urinating. He took spirits nitre and some simple remedies, but found no benefit.

*Symptoms on Examination.*—Pulse 110, tongue furred. He had passed no urine for thirty-six hours. There was great œdema of scrotum and penis; a brown discoloration extended from the under surface of penis along each side of raphe of scrotum for an inch, as if a slough was threatened.

An incision was at once made into the scrotum near an inch in depth—commencing near the perineum and terminating at the penis—on both sides of the raphe.

The walls of incision were bloodless and of a straw color. They presented the appearance of straw colored jelly divided. The pent up effusion began to flow rapidly, and was scarcely stained with blood. Penis was also strutted with effusion. Prepuce was punctured, and swelling in it somewhat diminished. Yet the penis remained rigid, swelled, and it was quite evident that a column of urine was pressing down upon some obstruction within the urethra.

A warm hip-bath was ordered to be repeated at short intervals and fomentations to be made to incisions.

12½ M.—Swelling of scrotum reduced more than half. No change in penis—prepuce had refilled. It was again punctured and an effort made to dislodge the obstruction, situated about three-fourths of an inch from the meatus urinarius. But owing to the great swelling and the very small meatus, it was impossible to use instruments to advantage.

I therefore determined to cut down upon the obstruction. As soon as the bistoury reached the urethra, a gush of urine took place, and the bladder was emptied, greatly to his relief. A calculus the size of a bean was extracted from urethra, which I here exhibit.

Ordered fomentations and poultices to be kept up and left an anodyne for the night.

5th, 10 A.M.—Passed restless night. Pulse 120, tongue furred, bowels moved. A small slough the size of a nickel had taken place in the integument at junction of penis and scrotum. He suffers from incontinence of urine, which flows through both openings. Very little swelling in scrotum. Ordered *milk diet.*

5 P.M.—Pulse 100. Continue fomentations or poultices.

6th, 10 A.M.—Fever arose in night. Pulse 120. Ordered quinine for to-night. Has more control over bladder. Urine passes freely through both openings. Continue poultices.

7th, 12 M.—Pulse 100. Slept well. Takes nourishment.

8th, 11 A.M.—No fever. Swelling of scrotum and penis nearly disappeared.

9th, 10th, 11th.—Doing well. Incisions into scrotum healing rapidly. Slough granulating.

12th—Restless last night. Pulse 110. Incision of urethra much contracted.

13th.—No fever. Good appetite.

18th.—Is up and going about. Only a few drops of water pass through incision.* Scrotum natural size—incisions into it healed over. Slough almost well.

* Incision into urethra closed June 1st.

*Case 2.—Œdema of Scrotum and Penis—Treated by Incisions—Recovery.*

HISTORY.—January 6th.—Wm. L., aged 43 years. Says he has stricture, and has, on several occasions, passed gravel (meaning calculi) from the bladder. Three days ago was taken with difficulty in attempting to urinate. Has passed none for twenty-four hours.

3 P. M.—Symptoms on examination. Pulse 105. Tongue furred. Scrotum and penis swelled. There was no swelling twenty-four hours ago. The scrotum has that peculiar semi-transparent, glossy appearance with a delicate pink hue. To the touch it reminds one of pressing on a muscular part.

Two incisions, one on each side of raphe, extending from the perineum to near the penis, were made. They were near an inch in depth. The sides of wounds resemble a mass of straw colored jelly. From them the serum, stained with blood, dripped rapidly, and in a short time the scrotum became soft to the touch.

The prepuce was punctured, and an incision through the integument of the dorsum of penis was made. It was only a short while before this organ was very much reduced in size also. Not being able to urinate, a catheter was resorted to, after some delay with what appeared to be a stricture, from the resistance — situated near the membranous portion — the instrument entered the bladder, and a copious discharge of urine took place. Ordered anodyne and poultice to genitals.

7th, 8 A.M.—Scrotum and penis less than half their size at operation. Pulse 110. Tongue furred. No appetite. Passed no urine. Catheter again used, but gave much pain. Ordered anodyne—beef essence.

6 P.M.—Catheter used with pain. Ordered anodyne for night.

8th, 9 A.M.—Pulse 120. There is pain and swelling in perineum. Urine drawn off with catheter. Scrotum and penis reduced three-fourths. Ordered milk punch.

6 P.M.—Urine drawn off.

9th, 8 A.M.—Pulse 118. Perineal swelling very much increased. Used catheter. Continue beef essence and egg-nog.

6½ P.M.—Used catheter. Gave anodyne.

10th, 8 A.M.—Pulse 120. Urine drawn off. Some discharge of pus—a few drops through catheter. Perineum projects more. Yet firm. Continue milk punch, etc.

5 P.M.—Urine drawn off, etc.

11th, 9 A.M.—Pulse 110. Catheter used and a little pus discharged. Perineum a little soft. A sound was introduced, and the perineal abscess opened. The discharge of pus was large, and very offensive.

On introducing the finger, found a small hole in the urethra near the membranous portion. Anodyne given, and supporting treatment ordered.

6 P.M.—Pulse 100. Passed urine, chiefly through perineum. Took more nourishment. Wounds in scrotum and penis nearly healed.

12th.—Pulse 95. Passed urine on yesterday. Appetite better.

13th.—Condition good. Walls of abscess contracted very much. Less urine through perineal opening.

14th.—Doing well.

15th and 16th.—Incisions into scrotum and penis are well.

17th.—Only a few drops of urine pass through artificial opening.

20th.—Water passes naturally.

I might give other cases of the same affection, presenting like symptoms, with the same treatment and results. But these suffice to present the disease in its true characters. They show its pathology—that it is a serous effusion into the cellular tissue situated between the tunica vaginalis and dartos. And they show further, I think, that, unless the already distended scrotum is relieved of the pressure from the constantly increasing effusion within, it will almost certainly slough. It would seem clear, then, that the remedy, from which most benefit is to be derived, lies in the free use of the bistoury.

